# Assessing the relationship between maternal and neonatal factors and low birth weight in Iran; a systematic review and meta-analysis

**Published:** 2015

**Authors:** J Bazyar, S Daliri, K Sayehmiri, A Karimi, A Delpisheh

**Affiliations:** * Student Research Committee, Faculty of Health, Ilam University of Medical Sciences, Ilam, Iran; ** Department of Biostatistics, Psychosocial Injury Prevention Research Center, Tehran University of Medical Sciences, Ilam, Iran; *** Faculty of Medicine, Ilam University of Medical Sciences, Ilam, Iran

**Keywords:** low birth weight, maternal and neonatal factors, meta-analysis, systematic review

## Abstract

**Introduction.** Low birth weight is an important indicator of the health of babies. A low birth weight is a leading health problem and a major reason for death in newborns. This study targeted to assess the relationship between maternal and infant factors and low birth weight in Iran through a systematic review and meta-analysis.

**Materials and Methods.** This paper was a systematic review and meta-analysis of the relationship between maternal/ infant factors and low birth weight based on the published research papers conducted in Iran. To achieve this goal, two trained researchers independently elicited all the relevant articles by using the appropriate keywords and their combinations in SID, Madlib, Iranmedex, Irandoc, Google Scholar, Pubmed, ISI, Scopus and Magiran databases. The results of the study were combined with SPSS 20 and STATA software.

**Results.** In the initial stage, 25 more relevant articles out of 46 papers were selected. The gestational age with less than 37 weeks and prenatal care had the most (CI: 27- 14. 53, OR: 19.81) and the least (CI: 1.86, OR: 1.5) effect on the low birth weight in newborns, respectively.

**Conclusion.** This study showed that there is a significant relationship between the low birth weight and multiple births, pre-eclampsia, maternal weight gaining during pregnancy, baby’s gender, and pregnancy age. Hence, controlling the factors above in mothers during pregnancy by the health authorities could lead to the birth of infants with a healthy weight and consequently the number of infants with low birth weight will decrease.

## Introduction

Nowadays, low birth weight is one of the most severe health problems all around the globe [**1**]. By low birth weight, we mean a birth weight of less than 2500 grand and by very low birth weight, we mean a birth weight of less than 1500 gr. Additionally, an immensely low birth weight is used for those infants with less than 1000 gr birth weight [**2**]. Due to their exceptional circumstances, infants with a low birth weight are not capable of adapting themselves outside the womb and are at a greater risk of mortality [**3**]. The mortality of infants with low and very low birth weight is respectively of 40 and 200 times more than that of the normal-weight newborns. In Iran, two-thirds of the infant deaths in the first 24 hours after birth, occur in infants with a low birth weight [**4**].

The prevalence of a low birth weight varies in different populations. According to the reports published by the World Health Organization (WHO) in 2000, totally 18 million children are born with a low birth weight per year, which is equal to 14% of all the births [**5**]. In Iran, this statistics is equal to 11.56 % [**6**]. A low birth weight in both advanced and developing countries imposes a tremendous pressure on the health care system and family members together [**7**]. Many factors affect the low birth weight, among others, genetic, environmental, embryonic, placental and maternal could be mentioned [**1**,**8**]. Also, the socioeconomic status, maternal age, ethnic factors, occupation, educational background of the mother and birth season seem to be influential in birth weight [**9**-**12**]. Based on several studies, the risk of low birth weight in mothers under 19 and over 35 has been confirmed [**13**]. Women over thirty-five years old are at high risk of maternal and fetal complications including obesity, chronic hypertension caused by pregnancy, etc., prenatal complications including preterm birth, stillbirth, low birth weight, intrauterine growth retardation, admission in the neonatal intensive care unit and congenital malformations [**14**]. 

Birth weight depends on many factors including genetic, biological, psychosocial, and environmental factors and different reports respect the effect on each of the factors on birth weight. Importantly, birth weight plays a driving role in the next periods of the baby’s life; therefore, detecting the factors associated with birth weight and revising some of the adjustable factors such as opting for a proper age for pregnancy and a healthy weight during pregnancy seem to be important. This research aimed to specify the impact of maternal and neonatal factors on low birth weight in Iran through a systematic review and meta-analysis. 

## Materials and methods

This study was a systematic review and meta-analysis on the relationship between maternal and infant factors and low birth weight in Iran. The results were acquired through all the relevant published articles in the national and international academic journals by the end of the year 2014 through searching in different databases including Madlib, SID, Scopus, Web of Science, ISI, Pubmed, Google Scholar, Irandoc, Iranmedex, and Magiran. The searching process was based on Persian key terms such as, low birth weight in babies, factors influencing low birth weight, low birth weight in Iran, preterm birth, multiple pregnancies, and pre-eclampsia of the mother, which were used individually, or in different combinations. Needless to say that in the external databases, their equivalents were used. The search of the articles was based on a clearly defined strategy and with the help of two well-trained researchers.

**The selection of the studies**

**Fig. 1:**
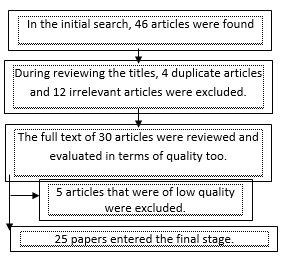
Flowchart details of entrance and selection stages for the systematic review and meta-analysis studies

All the items and dissertations relevant to low birth weight were studied until the end of the year 2014. The researches lacking sufficient information or those inaccessible were excluded from the study. To avoid bias, two well-trained researchers searched the articles independently. Based on this strategy, 46 relevant articles were found, among which four repetitive and 12 irrelevant items, which were excluded from the study. After reviewing the abstracts, five pieces lacked required information and thus were dropped. Finally, 25 articles met the required criteria and were included in the meta-analysis step (**Fig. 1**). To evaluate the quality level of the manuscripts, STROBE checklist was used [**15**]. 

**Data elicitation**

Data elicitation procedure was done through a pre-made list including sample size, the place, and date of the study, Odds ratio of the variables and the standard deviation. In cases in which the studies did not report the Odds ratio directly, this factor was estimated through calculating the related data to the Odds ratio. 

**The inclusion criteria**

All the Persian and English studies conducted in different regions of Iran regarding low birth weight (without any other complications) and those that gained the required score were included in the survey.

**The exclusion criteria**

After evaluation, the studies that did not gain a favorable rating, those conducted on a particular group or those that lacked a sufficient number of samples or samples of other diseases associated with the risk factors, were excluded from the study.

**Statistical analysis**

The statistical analysis of the data was done through the random pooled relative risk (effect method) and Review manager 4.2 software. In order to assess the amount of disparities among the results, I2 factor was used and for analyzing the data, SPSS20 and STATA software were also used. 

## Results

In this study, 46 articles based on searching in different databases were selected among which four duplicate and 12 irrelevant articles were excluded from the study. Finally, after reviewing the full text of 30 articles, 25 articles were included in the survey. The information regarding the selected items is presented in **Table 1**.

**Table 1:** General characteristics of the articles which were eligible for the study

Author	The place of the study	Date of publication	Author	The place of the study	Date of publication
Kouhdani, F [**16**]	Tehran	2010	Ranjbaran, M [**17**]	Markazi	2015
Delaram, M [**18**]	Shahrekord	2007	Mahmoodi, Z [**19**]	Tehran	2015
Eghbalian, F [**20**]	Hamedan	2007	Fadakar Soogheh, K [**21**]	Rasht	2012
Vahdaninia, M [**22**]	Tehran	2008	Tayebi, T [**23**]	Sari	2013
Taheri, F [**24**]	Birjand	2006	Davoudi, N [**25**]	Mashhad	2012
Roudbari [**26**]	Zahedan	2007	Bahrami, N [**27**]	-	2012
Sohrabi, D [**28**]	Zanjan	2007	Mirzarahimi, M [**29**]	Ardebil	2010
Jafari [**30**]	Zanjan	2010	Aramesh, M [**31**]	Ahvaz	2013
Rafati, Sh [**32**]	Tehran	2005	Shahri, P [**33**]	Ahvaz	2012
Karimian, S [**34**]	Qom	2002	Tabatabi, Sh [**35**]	Tehran	2010
Eslami, Z [**36**]	Yazd	2001	Eftaekhar, H [**37**]	Bandar Abbas	2007
Hajian, K [**38**]	Babol	1999	Mirzarahimi, M [**39**]	Ardebil	2009
			Tootoonchi, P [**40**]	Tehran	2007

The variables tested in this study included the relationship between preterm birth, infant gender, maternal weight gain during pregnancy, the mother’s prolificacy, infection due to preeclampsia and health care during pregnancy.

**Fig. 2:**
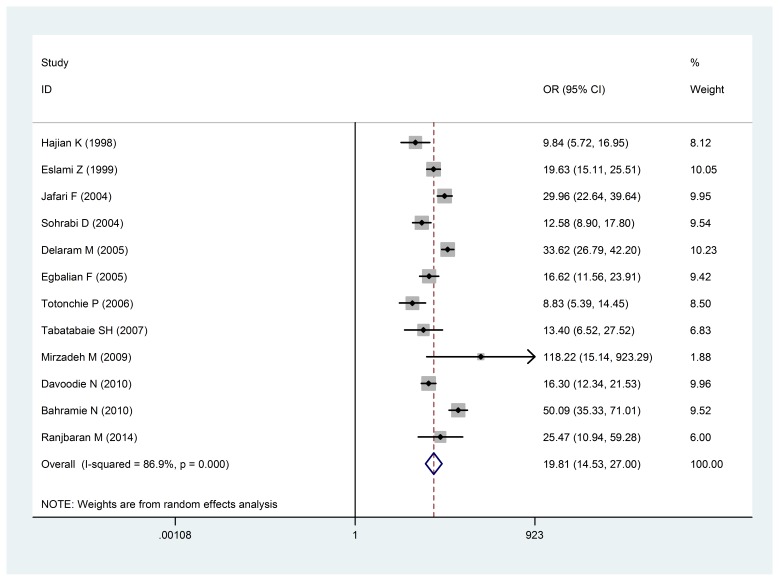
The Odds ratio of premature birth of less than 37 weeks with a low birth weight of a 95% confidence interval

The analysis of 12 studies that investigated the relationship between preterm birth and low birth weight was carried out in the country. The estimated odds ratio (OR) of LBW in women who had a preterm birth less of than 37 weeks, was 19.81 (14-27, confidence intervals: 95%), which indicated that the preterm birth could lead to an increase of low birth weight (**Fig. 2**). The dispersion test was also positive for the calculation (I286.9%: and P < 0/ 0001).

**Fig. 3:**
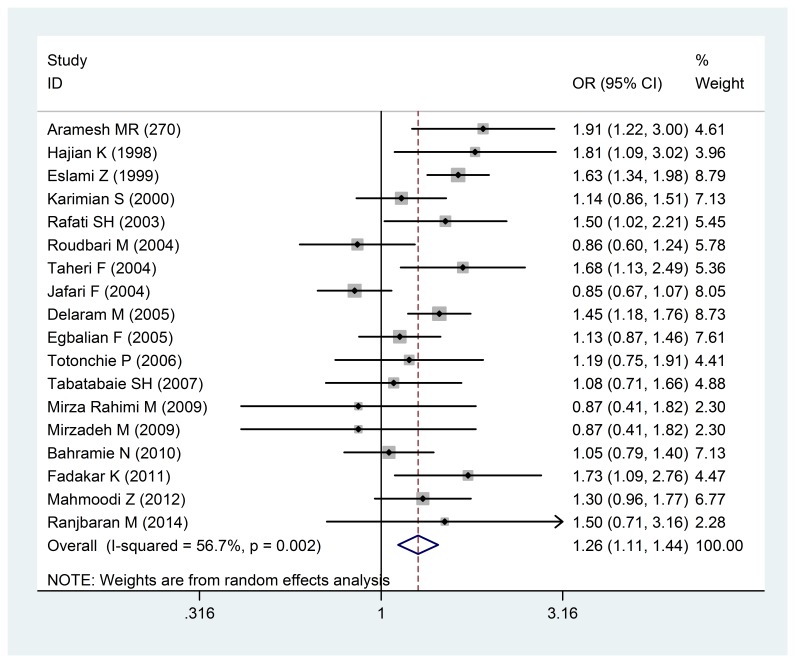
The Odds ratio of the infant’s gender with a low birth weight of a 95% confidence interval

The Odds ratio of premature birth of less than 37 weeks with a low birth weight of a 95% confidence interval in the studies reviewed was based on the date of publication and the author through random-effects model. The midpoint and the length of each segment indicated an Odds ratio and a 95% confidence interval in each case. The Diamond Mark showed the Odd ratio in all the studies, which indicated the relationship between the infant’s gender and the low birth weight. 18 studies conducted in Iran reviewed the odd ratio (OR) of low birth weight with female to male proportion, being estimated at 1.26 (1.44-1.11: 95% confidence interval) (**Fig. 3**), which statistically showed a significant relationship (I 2:56.7% and P = 0/ 002).

The Odds ratio of the infant’s gender with a low birth weight of a 95% confidence interval in the studies was reviewed based on the date of publication and the author through the random-effects model. The midpoint and the length of each segment indicated the Odds ratio and a 95% confidence interval in each case. The Diamond Mark showed the Odds ratio in all the studies. 

**Fig. 4:**
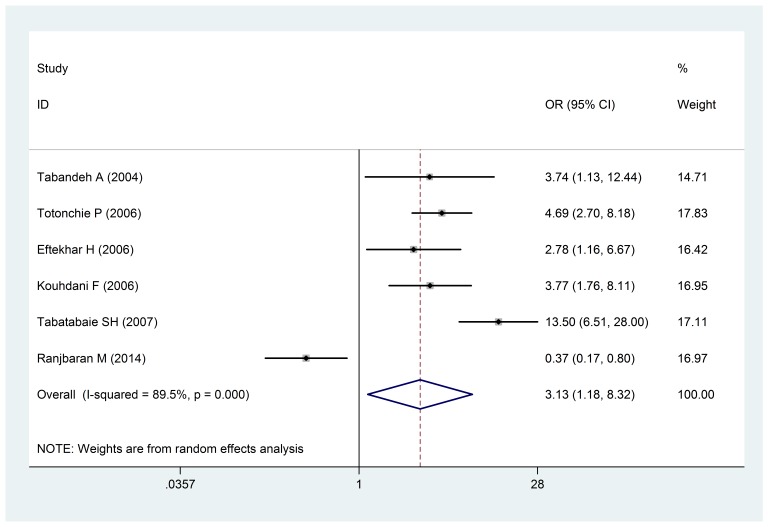
The Odds ratio of maternal weight gain during pregnancy and infants with a low birth weight of a 95% confidence interval

The risk of infants with a low birth weight in mothers who did not have an ideal weight during their pregnancy was estimated OR: 3.13 (1.18-8.32: 95% confidence interval) (**Fig. 4**). The dispersion test was also positive (I 2: 89.5% and P < 0.0001).

The Odds ratio of maternal weight gain during pregnancy and infants with a low birth weight of a 95% confidence interval in the studies was reviewed based on the date of publication and the author through the random-effects model. The midpoint and the length of each segment indicated the Odds ratio and a 95% confidence interval in each case. The Diamond Mark showed the Odds ratio in all the studies. 

**Fig. 5:**
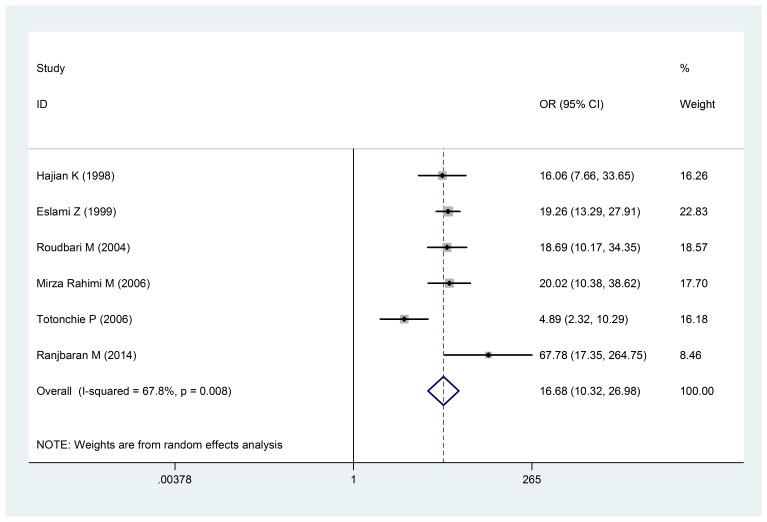
The Odds ratio of multiple births of mother and infants with a low birth weight of a 95% confidence interval

As far as the relationship between multiple birth and low weight, six articles were found in which the Odds ratio (OR) of infants with a low birth weight in multiple births was estimated at 16.68 (10.32-26.98: 95% confidence intervals) (**Fig. 5**). The dispersion test for this calculation was I 2: 67.8% and P < 0.008.

The Odds ratio of multiple births of mother and infants with a low birth weight of a 95% confidence interval in the studies was reviewed based on the date of publication and the author through the random-effects model. The midpoint and the length of each segment indicated the Odds ratio and a 95% confidence interval in each case. The Diamond Mark showed the Odds ratio in all the studies.

**Fig. 6:**
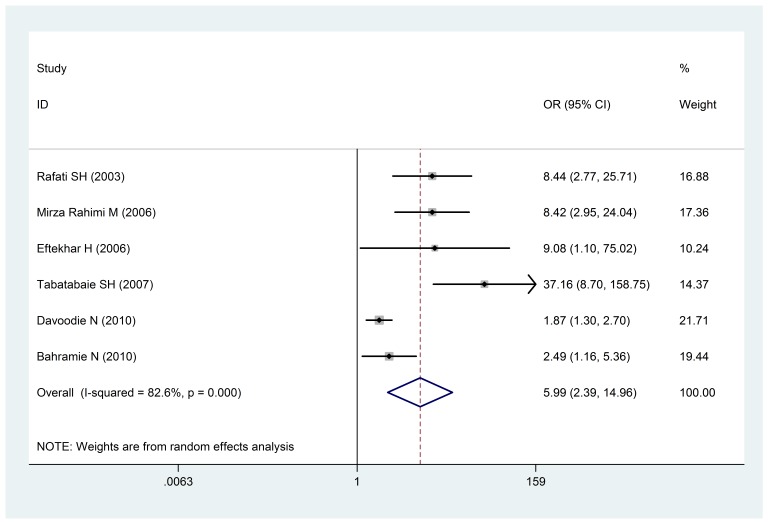
The odds ratio of mother pre-eclampsia and infants with a low birth weight of a 95% confidence interval

Based on six articles respecting the infection of mother to pre-eclampsia with a low birth weight, the Odds ratio of underweight infants birth in mothers infected to pre-eclampsia during their pregnancy was estimated at 5.99 (14.96- 2.39: confidence intervals 95%). The scattering test for these calculations was I 2: 82.6% and P < 0.0001, which showed a significant relationship (**Fig. 6**).

The odds ratio of the mother pre-eclampsia and infants with a low birth weight of a 95% confidence interval in the studies was reviewed based on the date of publication and the author through the random-effects model. The midpoint and the length of each segment indicated the Odds ratio and a 95% confidence interval in each case. The Diamond Mark showed the Odds ratio in all the studies.

**Fig. 7:**
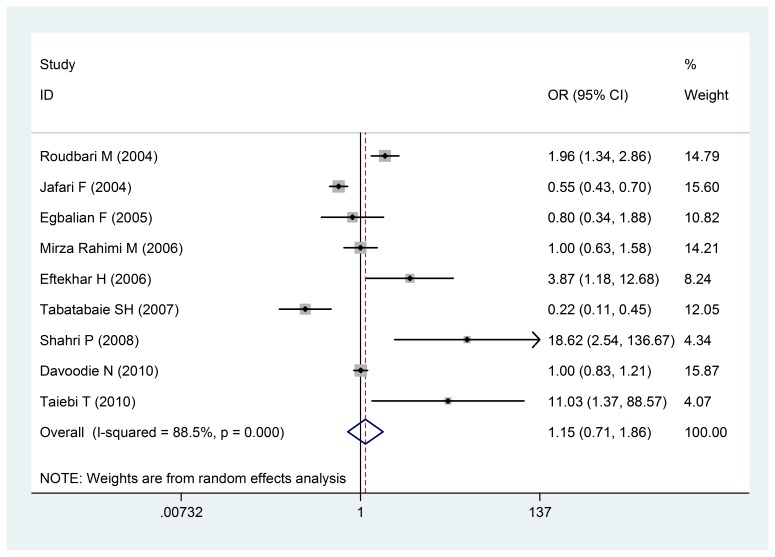
The odds ratio of the inadequacy of caring during pregnancy and infants with a low birth weight of a 95% confidence interval

By reviewing the relationship between the birth of underweight infants and prenatal care during pregnancy in mothers who had an inadequate care during pregnancy (less than six times), the following values were estimated OR: 1.15 (0.71- 1.86: confidence intervals 95%), that did not show a significant correlation between them (**Fig. 7**).

The odds ratio of the inadequacy of caring during pregnancy and infants with a low birth weight of a 95% confidence interval in the studies was reviewed based on the date of publication and the author through the random-effects model. The midpoint and the length of each segment indicated the Odds ratio and a 95% confidence interval in each case. The Diamond Mark showed the Odds ratio in all the studies.

## Discussion

In this meta-analysis study, 25 relevant articles including 11 case-control, 3 cohorts, and 11 cross-sectional studies were reviewed. In the assessment of the relationship between premature birth, 12 studies were perused. In order to study the infant’s gender, multiple births, mother infection to pre-eclampsia, caring during pregnancy, 18, 6, 6, nine articles were analyzed, respectively. Additionally, for the examination of the relationship between weight gain during pregnancy and low birth weight, totally six articles were reviewed. 

This study revealed that there was a strong correlation between premature birth and low birth weight, so that, infants of the mothers, who had a preterm birth, suffered a low birth weight of 19.8 times more than those children who were born at 37 weeks. In the study performed by Bahrami et al., 57.2 of the infants born through premature birth and only 2.6 of the children born through term birth were underweight [**27**]. In Ranjiran’s study done in Markazi province, 44.7 of the pre-term infants and 3.1 percent of the term infants suffered a low birth weight [**17**], which showed a significant difference, this fact supporting our findings in this study as well. 

In analyzing the relationship between the infant’s genders, the low birth weight was more common amongst female than male children. In a study conducted in Japan, a significant association among girls and low birth weight was observed [**41**], and it was also discovered that the mean of birth weight in male infants was more in comparison with the one of female children [**42**,**43**].

This research showed that the mother’s improper weight gain during pregnancy might lead to an increase in the number of underweight infants, so that the number of underweight babies in mothers who gained 6 kg during pregnancy was 3.3 times more than those parents who acquired a proper weight during pregnancy. In the conducted researches, a significant relationship was found between the mother’s weight gain and the low birth weight [**44**,**45**], that being, the mean number of infants in mothers with proper weight gain was more than of those with an improper weight gain [**46**,**47**].

**Limitations**

Some of the limitations that we faced with during the study included the lack of access to the full text of some of the studies, sufficient information in the studies, access to some unpublished manuscripts and insufficiency of samples in some of the studies.

**Concluding remarks**

The present paper was a systematic review and meta-analysis study that assessed the relationship between maternal and infant factors with a low birth weight in Iran. This study revealed that maternal factors including multiple delivery and premature birth have a great impact on the birth of such infants. There was a significant relationship between the gaining of a proper weight in mothers and the infection of mother to pre-eclampsia. Based on the finding of the study, health and care authorities are recommended to train parents in the respective fields and adopt the appropriate ways to protect mothers against the risk of a low birth weight.

**Acknowledgments**

The authors would like to thank the Research Center and the Student Research Committee of Medical Science University of Ilam for their help in conducting this research. 
